# Primary flap reconstruction of tissue defects after sarcoma surgery enables curative treatment with acceptable functional results: a 7-year review

**DOI:** 10.1186/s12893-015-0060-y

**Published:** 2015-06-09

**Authors:** Jenny Fabiola López, Kristiina Elisa Hietanen, Ilkka Santeri Kaartinen, Minna Tellervo Kääriäinen, Toni-Karri Pakarinen, Minna Laitinen, Hannu Kuokkanen

**Affiliations:** Department of Plastic Surgery, Unit of Musculoskeletal Diseases, Tampere University Hospital, Pirkanmaa Hospital District, Teiskontie 35, PO BOX 2000, Tampere, 33521 Finland; Department of Orthopedics and Trauma, Unit of Musculoskeletal Diseases, Tampere University Hospital, Tampere, Finland

**Keywords:** Sarcoma, Characteristics, Defect size, Flap reconstruction, Survival

## Abstract

**Background:**

Sarcomas, a heterogeneous group of tumors, are challenging to treat and require multidisciplinary cooperation and planning. We analyzed the efficacy of flap reconstruction in patients with bone and soft tissue sarcoma.

**Methods:**

Patient charts and operative records were retrospectively reviewed from January 2006 through October 2013 to identify sarcoma patient characteristics, postoperative complications, revisions, recurrences, and survival. Pedicled and/or free flap reconstruction was performed in 109 patients. Flap selection was based on defect size, and exposure of anatomically critical structures or major orthopedic implants.

**Results:**

Of 109 patients, 71 (65.1 %) were men, and mean age was 56.4 years. Tumors most frequently located in a lower extremity (38.7 %). Primary sarcomas comprised 79.2 % and recurrences occurred in 18.9 %. Wide resection was performed for 65.7 %, and there were 10 planned amputations combined with flap reconstruction. A total of 111 tumors received 128 flaps: 76 pedicled flaps, 42 free flaps, and 5 combined (10 total) pedicled + free-flaps. The success rate was 94 % for the pedicled flap group, 97 % for the free-flap group, and 100 % for the pedicle + free-flap group. Of 35 patients, 5 developed deep prosthetic infections. Only one amputation due to disease progression was performed. Satisfactory functional outcome was achieved in 69 %. Survival rate during a mean (standard deviation) 3(2) year follow-up was 83.5 %.

**Conclusions:**

Primary flap reconstruction after sarcoma surgery satisfies oncologic goals. Large tumors in difficult areas can be removed and complete tumor resection achieved. Our findings indicate a high survival rate after sarcoma surgery utilizing flap reconstruction and a low recurrence rate.

## Background

Soft tissue and bone sarcomas have characteristic patterns of biologic behavior on which staging and treatment protocols are based [1]. The international incidence rate ranges from 1.8-5 per 100,000 persons/year, with 11,410 new cases in 2013 [2]. The Finnish Cancer Registry reported an incidence of 0.7 and 1.4/100.000 persons/year for bone and soft tissue cancers [3].

Advances in the multimodal treatment approach of sarcoma patients have drastically improved survival and quality of life [4, 5]. Combined wide excision and adjuvant therapy remains the standard for local control without increased recurrence or mortality [6]. Nonetheless, recent studies demonstrated that narrower margins yield similar outcomes and complete tumor resection is the main aim for oncology surgeons [7–9].

Significant tissue defects may result from sarcoma excisions, with exposure of tendons, bones, joints, vessels, and prosthetic material, making substantial coverage crucial. Sarcoma patients require reconstructive procedures that provide quality vascularized tissue with a brief healing period, thus enabling adjuvant therapy.

Pedicled flaps are used most commonly to cover sarcoma defects and free flaps have gained popularity and provide fast healing, adequate functional results, and vascularized tissue coverage [10–12].

The benefits of primary tissue reconstruction for sarcoma patients, the most common postoperative complications, and the revision percentages remain unclear. The aim of this retrospective study was to analyze the results and benefits of primary tissue reconstruction in our multidisciplinary Sarcoma Unit.

## Methods

The Tampere University Hospital Sarcoma Unit registry identified a total of 359 sarcoma patients from January 2006 through October 2013. This research was approved by the Tampere University Hospital Research Committee and a patient photography release consent form was obtained.

Flap reconstruction was performed in 109 of these patients. Patient charts were reviewed to determine demographic characteristics, comorbidities, pertinent laboratory results, and body mass index (BMI). Tumor information (location, pathology, and grade) was also collected. Surgical data included: resection type, hardware used, and defect size. Flap choice included tumor/defect size (defined through pathology specimen as length x width), tissue type, function, and donor site availability, among others. There were three reconstructive options: pedicled flaps, free flaps, and pedicled + free (as two separate) flaps. Pedicled flaps in our study were defined as true perforator flaps, such as propeller flaps, and local broad-based fasciocutaneous or muscle flaps. Using a two-team approach, the operating time included tumor resection, fixation, and reconstruction. Outcome analyses of postoperative complications and revisions were calculated. Early complications were defined as those occurring within the first 30 postoperative days. Postoperative pathologic margins were studied, as well as patient functionality. Finally, disease recurrence, progression, and patient survival were analyzed.

Data are presented as the mean (standard deviation) unless otherwise noted. Statistical analysis was performed using SPSS® version 22. For continuous variables, p values were calculated using the Kruskal-Wallis non-parametric test, and for the categorical variables, Fisher’s exact test was used. Box plots were applied for defect size analysis, and the Kaplan-Meier test was used to analyze patient survival with Cox regression for individual variable analysis.

## Results

A total of 109 sarcoma patients that underwent reconstruction were identified. Demographic information concerning comorbidities, age, BMI, and laboratory tests is provided in Table 1. The majority of patients (71, 65 %) were men. Mean patient age was 56.4 years and mean BMI was 26.22 kg/m^2^. Age was significantly higher in the pedicled group and lower in the pedicled + flap group. The remaining demographic characteristics were not significantly different among the study groups.

**Table 1:** Patient Characteristics

	Number (%)
Sex	
-Women	38 (34.8 %)
-Men	71 (65.1 %)
Comorbidities	
-Smoking	14 (12.8 %)
-Heart condition	27 (24.7 %)
-Diabetes	14 (12.8 %)
-Hypertension	38 (34.8 %)
Age (yr)	56.4 (20.5)
BMI (kg/m2)	26.2 (4.8)
Laboratory	
-Hb (g/L)	131 (18.7)
-WBC (mm^3^)	7689 (2989)
-Platelets (μL)	293,981 (96997)
-Na (mEq/L)	138.9 (2.9)
-K (mEq/L)	3.95 (0.3)
-Creatinine (mg/dl)	0.42 (0.1)
-GFR (mL/min/1.73 m^2^)	233.4 (105)
-INR (SU)	1.12 (0.1)

BMI (body mass index), Hb (hemoglobin), WBC (white blood count), Na (sodium), K (potassium), GFR (glomerular filtration rate), INR (international normalized ratio). Values expressed in number (n) and percentages (%) for categorical variables; and mean and standard deviation (SD) for continuous variables

Tumor localization and presentation (primary, recurrent, and metastatic) are shown in Table 2. There were more free-flap reconstructions for lower limbs and more pedicled flap reconstructions for thoracodorsal defects. Pedicled + free flaps were used more often for lower trunk reconstruction (*p* = 0.02). Lower trunk tumors were predominantly located in the pelvic and sacral regions. Free and pedicled flaps were used equally for head and neck, and lower trunk reconstruction. In the upper extremities, pedicled flaps were common, due to the versatility of pedicled radial forearm flaps.

**Table 2:** Tumor localization and presentation

Tumor Characteristics	Number (%)
Localization	
-Head and neck	8 (7,20 %)
-Thoracodorsal	23 (20,72 %)
-Abdominopelvic/ lumbosacral	27 (24,32 %)
-Upper extremity	10 (9,00 %)
-Lower extremity	43 (38,73 %)
Primary^a^	88 (79.2 %)
Recurrence	21 (18.9 %)
Metastasis	2 (1.8 %)

^a^2 patients had new primary tumors from distant sites

The pathologic distribution of the tumors in this study is shown in Table 3. High grade tumors comprised 60.3 %, low grade 22.5 %, and intermediate 8.1 % (9 % not reported). Neoadjuvant therapy was performed in 16 patients (14 chemotherapy and 2 radiotherapy). Adjuvant therapy was distributed as follows: 22 patients received only chemotherapy, 23 received only radiotherapy, and 21 received both chemotherapy and radiotherapy. According to Enneking’s classification, surgical resections were performed as follows: wide (66 %), marginal (22 %), radical (8 %), and intralesional (3.6 %) [5]. In our series, planned pathologic wide margins were achieved in 94 % and planned marginal resections were accomplished in 96 %. This was possible despite the fact that 69 % of the tumors were T2 (>5 cm). Our multidisciplinary sarcoma team follows the Scandinavian Sarcoma Group Guidelines (SSG), which originally defined ‘the wide cuff of healthy tissue’ as 5 cm [8]. There has been a gradual shift towards accepting narrower margins that led the SSG to redefine wide margins as a ‘cuff of 10 mm non-fascial tissue surrounding the tumor’. This opinion has gradually changed to accept even finer margins [8, 9]. In our series, R0 resections were obtained in 85 % of cases. Under the SSG policy, adjuvant radiotherapy is indicated if the margins are narrow and for all high-grade soft tissue sarcomas (STS) greater than 8 cm in size [9]. Additional surgery is not recommended unless there is a local recurrence after adjuvant radiotherapy. Adjuvant chemotherapy is given based on tumor grade, histology, age, and tumor size, and not based on the margins [13].

**Table 3:** Sarcoma pathology

Sarcoma Pathology	Number (%)
Rabdomyosarcoma	2 (1.8 %)
Ewing	3 (2.7 %)
MPNST	3 (2.7 %)
Synovial sarcoma	6 (5.4 %)
Hemagio-angiosarcoma	7 (6.3 %)
Dermatofibrosarcoma/fibrosarcoma	9 (8.1 %)
Osteosarcoma	12 (10.8 %)
Leiomyosarcoma	12 (10.8 %)
Liposarcoma	17 (15.3 %)
Chondrosarcoma	19 (17.1 %)
Undifferentiated Pleomorphic	21 (18.9 %)
TOTAL	111 (100 %)

^a^Values are expressed as number and percentage. MNSPT (malignant peripheral nerve sheath tumor)

Reconstruction with metal implants was used for 29 % of the patients (endoprostheses, plates, and screws or spinal instruments) and surgical meshes for 18 % of the patients. Endoprosthetics were used in 14 patients. Autologous non-vascularized bone was used at the beginning of the study in three patients, but was replaced with vascularized bone due its clear benefits. The risk for infection was significant in patients who underwent endoprosthetic placement and bone grafting. In our study, muscular flaps were the preferred choice for prosthetic material coverage as they serve as an ideal vascular tissue barrier. In our experience, coverage with highly vascular tissue such as muscle is a safe method of protecting against infection and fistula formation. Additionally, muscular tissue provides volume to fill the dead space [14–17].

**Fig. 1:**
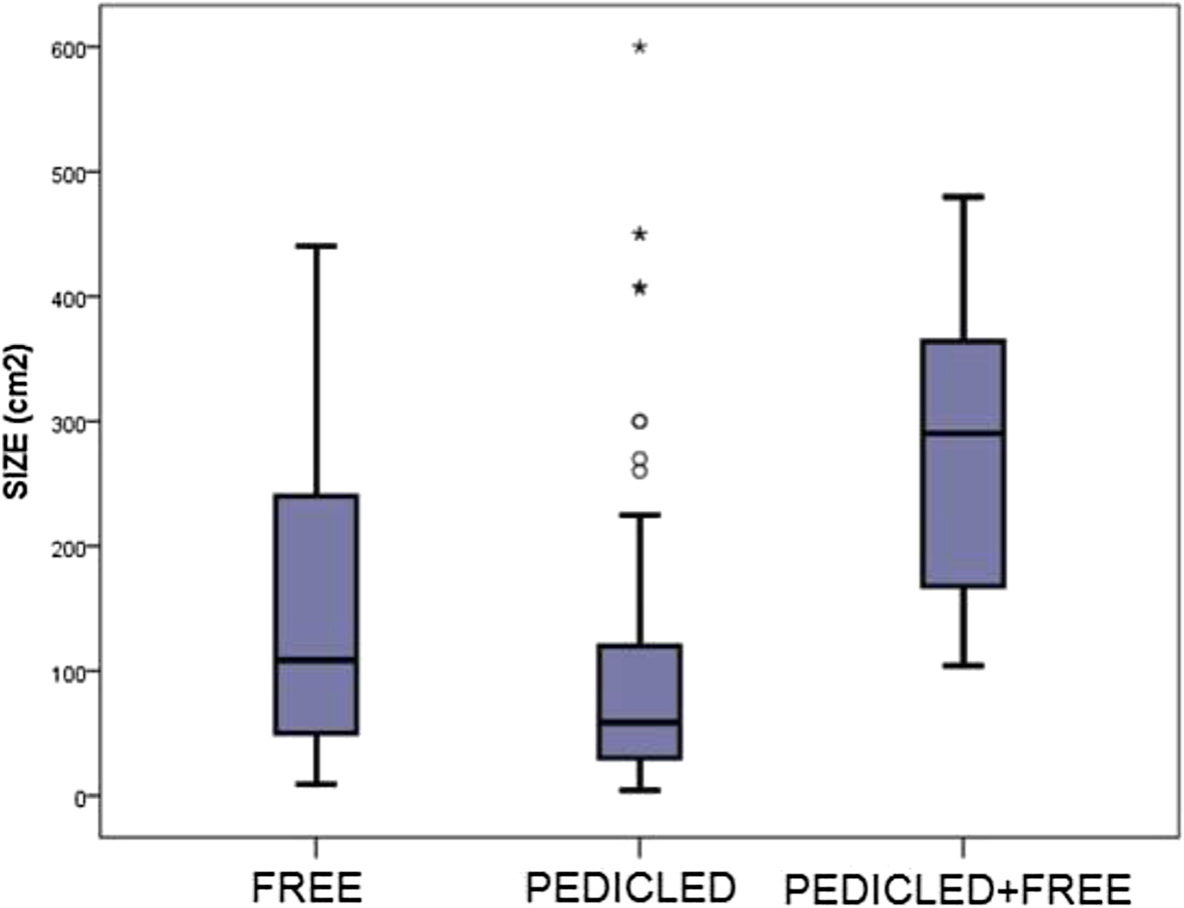
Sarcoma Defect Size Distribution. Box plot showing the distribution of flap size among the reconstructive groups. Double flap reconstructions were required for larger defect sizes (*p* = 0.004)

From a total of 111 defects, the distribution of the soft-tissue defect size varied from 4 cm [2] to 600 cm^2^ (Fig. 1). Larger defects required combined reconstructions (*p* = 0.004).

Of 128 flaps, 42 were free, 76 pedicled, and 5 combined pedicled + free (10 total). The flaps comprised 39 % musculocutaneous, 36.5 % muscular, 18.6 % fasciocutaneous, and 5.6 % osteofasciocutaneous. The latissimus dorsi (LD) flap was most commonly performed in the free-flap group (62 %), 4 of which were neurovascular and 2 as a chimeric flap with scapular bone. An endoscopic LD harvest was performed in one case. An innervated (functional) free gracilis flap was also used for a brachial defect after a failed pedicled flap (Table 4).

**Table 4:** Flap characteristics: flap type

Flap	Pedicled	Free
Latissimus dorsi	22 (27.1 %)	29 (61.7 %)
ALT	2 (2.4 %)	4 (8.5 %)
ALT + vastus lateralis	2 (2.4 %)	4 (8.5 %)
Vastus lateralis	5 (6.1 %)	1 (2.1 %)
Radial forearm	3 (3.7 %)	2 (4.2 %)
Fibula	1 (1.2 %)	3 (6.3 %)
Rectus abdominis TRAM/VRAM	3 (3.7 %)	1 (2.1 %)
Gracilis neurovascular	-	1 (2.1 %)
Filet Flap	-	2 (4.2 %)
Gluteus	9 (11.1 %)	-
Gastrocnemius	14 (17.2 %)	-
Local fasciocutaneous	11 (13.5 %)	-
Trapezius	1 (1.2 %)	-
Crista iliaca	1 (1.2 %)	-
Tensor fascia lata	2 (2.4 %)	-
Sartorius	1 (1.2 %)	-
Soleus	1 (1.2 %)	-
Biceps femoris	1 (1.2 %)	-
Pectoralis	2 (2.4 %)	-
TOTAL	81 (100 %)	47 (100 %)

^a^Values are expressed as number and percentage. ALT (anterolateral thigh), TRAM/VRAM (transverse/vertical rectus abdominis myocutaneous)

The global mean operating time was 5.6(2.4) hr; 4.7(2.4) hr for the pedicled flap group (range 1–10.5), 6.6(1.8) hr for the free-flap group (2.5-11), and 7.7(0.8) hr for the pedicled + free flap group. Surgical time was greater in patients requiring two flaps (*p* = 0.001). Operative time was significantly lower for pedicled reconstructions (*p* = <0.0001 compared with free-flap reconstruction). In 11 patients, delayed reconstruction was performed due to patient condition and/or prolonged tumor resection.

The most frequent early complications encountered were: minor necrosis (30 %), delayed wound healing (17 %), infection (15 %), hematoma (10 %), seroma (8 %), and total flap loss (4 %). The incidence of pedicled and free-flap complications was not significantly different. The influence of comorbidities on early complications was not significant, except in total flap loss where cardiac disease appeared to have a significant role (*p* = 0.012). From a total of 35 patients who underwent prosthetic material placement, 5 suffered deep infections, and 3 required implant removal. One mesh required removal as a result of deep infection.

Wound healing complications were observed following neoadjuvant chemo- or radiotherapy. Of 14 patients receiving neoadjuvant chemotherapy, 6 reported wound complications such as infections and necrosis that delayed wound healing and required revision surgery (*p* = 0.10). Both patients receiving preoperative radiotherapy presented with complications of recurrent hematoma/seroma formation and infection that required revision surgery (*p* = 0.05).

The revision rates were as follows: 17 % for the pedicled, 13 % for the free, and 4 % for the pedicled + free-flap group. Wound revision for lavage and closure was significantly greater for the pedicled + free-flap group (*p* = 0.033). In the free-flap group, four patients required revisions due to vascular compromise (two required vein and two arterial and venous re-anastomosis); three with reported flap survival. The overall revision differences between the study groups and the number of revisions per patient were not significant (*p* = 0.086 and 0.116).

There was one total flap loss in the free-flap group (97 % survival); 4 total flap losses for the pedicled flap group (94 % survival), and no flap loss in the pedicled + free flap group. The pedicled + free-flap group had significantly more hematomas and wound healing problems than the other groups. The reason for this was the larger defect size in patients receiving both a pedicled and a free-flap reconstruction. When total flap loss was encountered, reconstruction was performed with new free flaps in two cases, with pedicled flaps in two cases, and with negative wound pressure therapy and multiple revision surgery followed by skin grafting in one case. The latter patient required fat grafting for volume replacement. Systemic complications were distributed equally among the groups and included pulmonary, cardiac, thromboembolic events, sepsis, and renal failure.

**Fig. 2:**
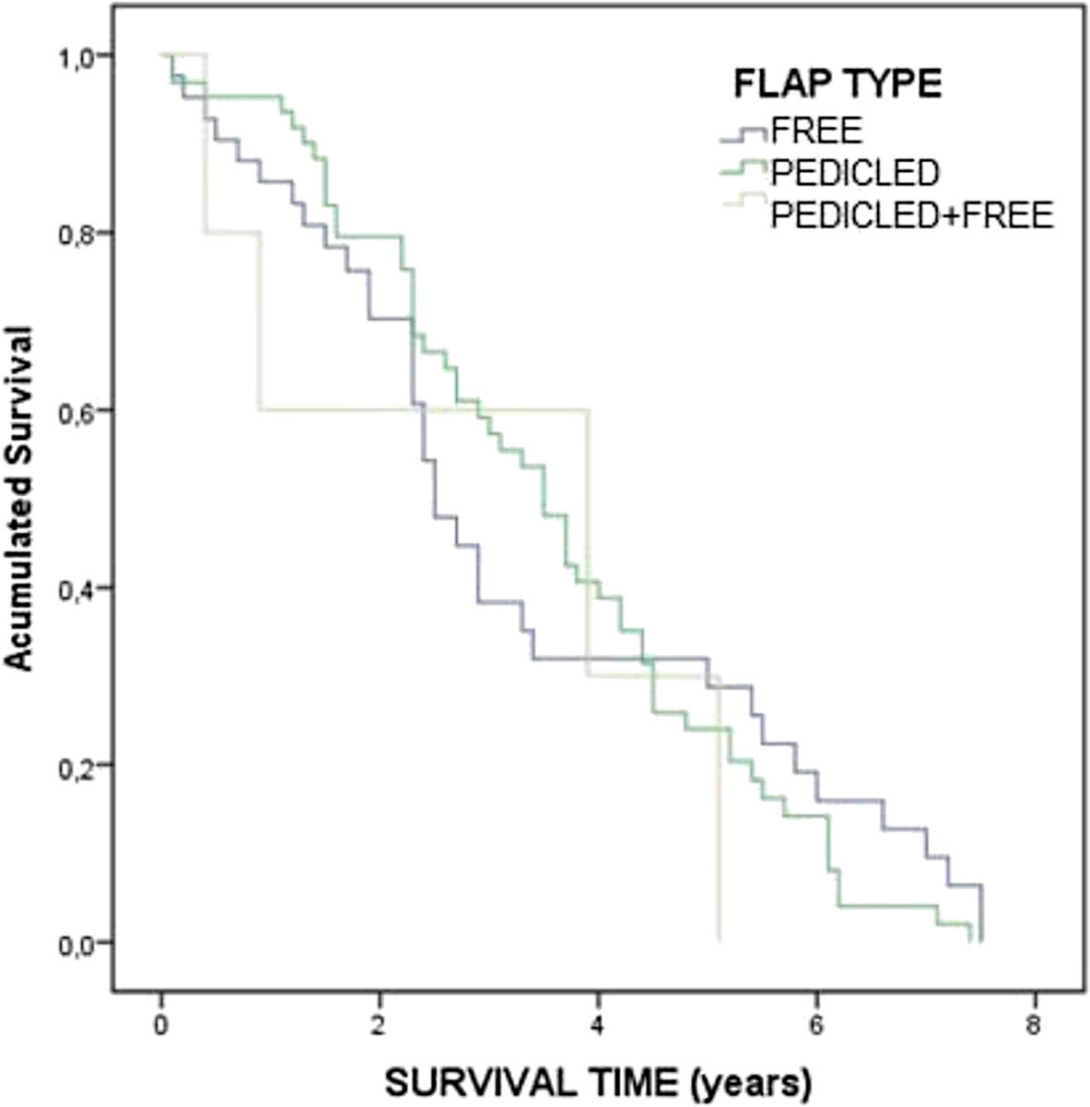
The Kaplan-Meier survival curve. Survival did not differ significantly among the three reconstructive groups during the study period

Local recurrence was 19.3 % and overall survival was 83.5 % (mean [standard deviation] follow-up time 3(2) years, range 0.1-7.7 years). Analysis of the correlation between surgical resection and recurrence revealed that 16/73 (22 %) of the patients with wide resections, 3/24 (18 %) with marginal resections, 2/4 (50 %) with intralesional resections, and 1/10 (10 %) with radical resections had a recurrence. The recurrence rate did not differ significantly among the study groups. Using the American Joint Committee on Cancer TNM clinical-pathologic staging for sarcomas, 28 % were stage I (9 % IA, 19 % IB), 29 % were stage II (15 % IIA, 14 % IIB), and 21 % stage III, for tumors that could be staged; [1] 24 patients (22 %) were considered stage IV. The common sites for tumor metastasis were lung (70 %), bone (15 %), liver (7 %), groin (7 %), and pelvis (4 %). One of the patients had disease progression that led to extremity amputation. Two early deaths were reported (within the first month of surgery). Survival ratios are provided in Fig. 2, and no significant differences were detected in the reconstruction groups. Sarcoma tumor recurrences significantly affected survival, with an 89 % risk of death.

The value of plastic surgery reconstruction was demonstrated in our series. Twelve patients would have been considered inoperable if flap reconstruction was not available. Similarly, without flap reconstruction, 19 patients would have been considered candidates for amputation. Distal limb tumors, tumors resected after whoops procedures, and axial tumors that required flap coverage due to defect size and vital organ exposure benefited the most from reconstructive surgery.

**Fig. 3:**
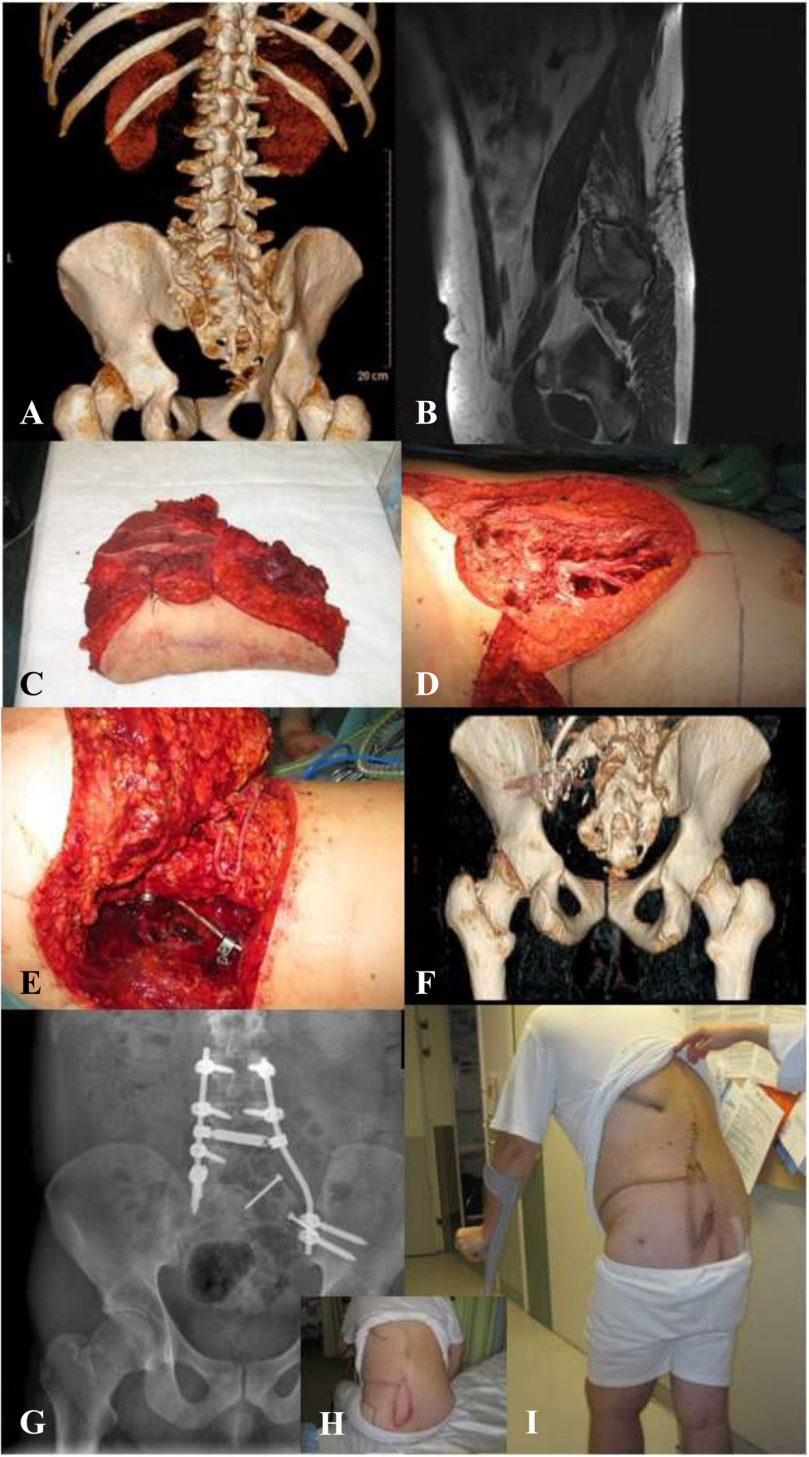
Patient Photographs. 31 year-old male with left ilium chondrosarcoma, and resection of the medial ilium, partial sacrum, and LV (5^th^ lumbar vertebra) hemicorporectomy, fixation and reconstruction with an osteomyocutaneous latissimus dorsi free-flap and saphenous vein transposition. **a,b** Preoperative images. **c,d** Biopsy specimen and defect. **e** Venous transposition. **f,g** Postoperative images. **h,i** Patient flap progression

**Fig. 4:**
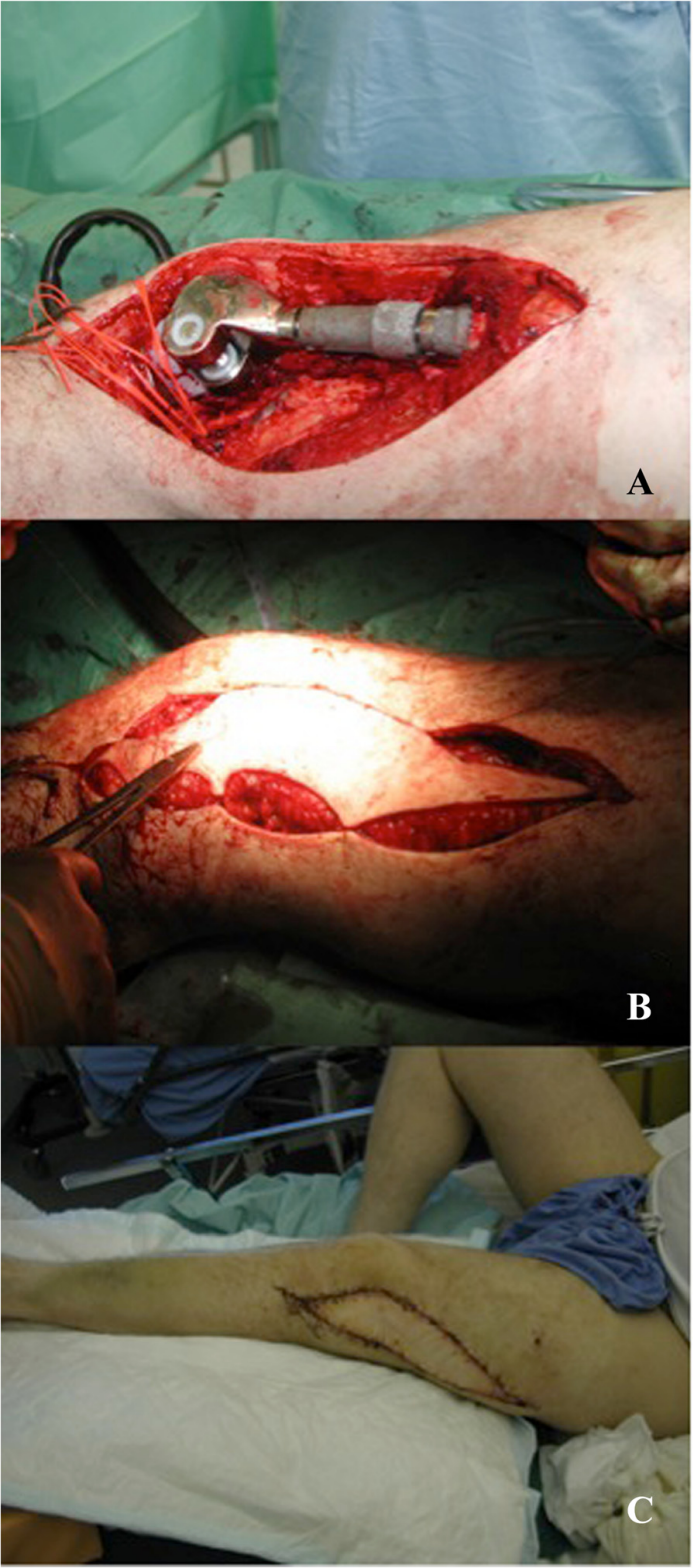
**a,b,c**. Patient Photographs. Example of the value of plastic surgery. Patient with megaprosthesis of the knee after sarcoma resection and the resulting defect. Reconstruction with ALT free flap and results

In our series, 96 % of the lower limb and trunk patients were able to ambulate after sarcoma resection and reconstruction, 41 % of these with assisted ambulation or with minor gait disorder. Considering that 27 % of the tumors involved the spine, sacrum, or pelvis axis, and that 39 % involved a lower extremity, functionality was considered adequate Figures 3 and 4 include examples fo hard and soft tissue sarcomas treated in our Department.

## Discussion

The goal of primary tissue reconstruction after sarcoma resection is to reconstruct the excised tissue and secure wound healing by filling the dead space. The possibility of primary tissue reconstruction allows the orthopedic oncologist to proceed freely with complete tumor ablation without worrying about wound closure. The SSG guidelines have gradually shifted to accept narrower margins. The patients studied here, however, were operated on at a time when the aim was to achieve wide margin of at least 10 mm of nonfascial tissue. This aim led to extensive resections, as reported here. Although wide margins remain the standard resection for sarcomas, narrower margins have demonstrated comparable outcomes based on longer follow-up and survival numbers [18, 19].

Marginal resections provide similar survival rates compared with wide resections [8, 9, 13, 18, 19]. Intralesional resection yields the highest numbers of recurrence at surgical margins [20]. In our study, the greatest percentage of recurrence was detected in patients undergoing intralesional resections, followed by wide, marginal, and radical, but the differences were not statistically significant. We attribute this result to the remarkably low number of intralesional resections performed compared with the wide or marginal resections.

Although neoadjuvant chemo- and radiotherapy negatively affect wound healing, the effects were not significant. The very low number of patients receiving neoadjuvant therapy may have influenced this result. STS, the most common type of sarcoma, is resistant to chemotherapy and therefore not commonly used in these patients. Patients with STS are usually older than those with bone sarcoma and chemotherapeutic agents for sarcoma treatment are highly toxic. Therefore, the benefits of chemotherapy are lower than the possible toxic complication rates in this group.

Adequate wound healing is mandatory for postoperative adjuvant therapy [20–22]. In the past, large tumor resections and reconstructions were avoided due to the detrimental effects of adjuvant therapy, patient morbidity, and high recurrence rate. Nonetheless, tissue transfer from unirradiated sites can overcome the detrimental effects of adjuvant therapy on wound healing [20–22]. Sarcoma reconstruction is associated with a high proportion of postoperative complications, likely due to the prolonged surgical time as well as extended postoperative bed rest and immunosuppressive therapies. In the present study, we found that creating two flaps significantly prolonged the operating time. The operating time was significantly shorter for pedicled flaps, compared with free flaps. The main reason for this result, however, is that free-flap surgical time included local options, such as transposition flaps. Cordeiro described major (12 %) and minor complications (7 %) following free-flap reconstruction, reporting flap success in 96 % of cases [12]. In our study, the number of early complications was 17 %, but only 20 % of the patients required revision surgery. Pedicled flaps are commonly considered more reliable and successful for oncologic cases, but we found that the free-flap success rate was equivalent. Our current preference for the use of free flaps in certain cases is due to the advantage of freedom of flap positioning, incorporating healthy vascularized tissue, avoiding kinking or stretching of the vascular pedicle, and quick healing periods. Adding volume and a protective barrier to oncologic defects using reconstructive flaps provides greater tolerance for subsequent adjuvant therapy [23–28].

Recurrences are considered high after flap reconstruction in the setting of advanced and high-grade sarcomas [24]. Initial presentation with recurrent disease and tumor size are important risk factors for subsequent recurrence [20–22, 24, 29, 30]. In our series, planned margins were achieved in over 94 % of the cases, even for large tumors. Overall patient survival was 83.5 % for the study period, which is higher than that reported previously, ranging from 50 % to 80 % [1–3, 31–34]. Sarcoma recurrence is reported to be ~25 % to 60 % [1, 2, 31–34]. In our study, recurrence was 19 %, slightly lower than that in previous reports. Flap reconstruction allows for complete tumor excision, and thus results in a good survival rate.

Proper flap selection is crucial in sarcoma defect reconstruction [29, 30, 35]. In general, free and pedicled flaps were equally effective for defect coverage. The limitations of the current study are the retrospective nature of the research and the heterogeneity of the study population. Additionally, the condition of each patient was unique, including tumor type and indication, making their reconstruction almost impossible to compare.

## Conclusions

Primary flap reconstructions after sarcoma surgery are often performed in our center for various reasons. The possibility for soft tissue and bone reconstruction allows the oncologic surgeon to perform a complete tumor excision with adequate margins [5]. Primary flap reconstruction also helps to protect orthopedic megaimplants and avoid deep infection. Additionally, an important aim is to maintain maximum patient function without compromising surgical oncologic principles. Enabling a patient to walk and return to daily activities of living after sarcoma resection vastly improves the patient’s quality of life. The availability of plastic surgery reconstructions enables surgery to be performed in some patients with complex cases, and can aid in preventing amputations in some cases.

Performing primary flap reconstruction contributes to satisfy the primary goal of sarcoma resection. The choice of the reconstructive method applied is profoundly individualized. Therapeutic planning for sarcoma patients should be accomplished by a multidisciplinary team involving oncologists, radiologists, pathologists, orthopedic oncologists, arthroplastic and spine surgeons, and reconstructive plastic surgeons.
